# Similarity of Phenotype in Three Male Patients With the c.320A>G Variant in *ALG13*: Possible Genotype–Phenotype Correlation

**DOI:** 10.1002/mgg3.70010

**Published:** 2024-09-23

**Authors:** Rebecca Finnegan, Mary O'Regan, Máire White, Gianpiero L. Cavalleri, Norman Delanty, Katherine A. Benson, Marie T. Greally

**Affiliations:** ^1^ Department of Paediatric Neurology Children's Health Ireland at Crumlin Dublin Ireland; ^2^ School of Pharmacy and Biomolecular Sciences Royal College of Surgeons in Ireland Dublin Ireland; ^3^ FutureNeuro Research Centre Royal College of Surgeons in Ireland Dublin Ireland; ^4^ Department of Neurology Beaumont Hospital Dublin Ireland; ^5^ Department of Clinical Genetics Children's Health Ireland at Crumlin Dublin Ireland

**Keywords:** *ALG13* gene, ALG13‐CDG, DEE36, genotype–phenotype correlation, X‐chromosome inactivation

## Abstract

**Background:**

Congenital disorders of glycosylation (CDG) are a group of neurometabolic diseases that result from genetic defects in the glycosylation of proteins and/or lipids. Multiple pathogenic genes contribute to the varying reported phenotypes of individuals with CDG‐1 syndromes, most of which are inherited as autosomal recessive traits, although X‐linked inheritance has also been reported. Pathogenic variants in the asparagine‐linked glycosylation 13 homolog (*ALG13*) gene have been implicated in the aetiology of developmental and epileptic encephalopathy (DEE) 36 (OMIM:*300776, DEE36). The NM_001099922.3:c.320A>G; p.(Asn107Ser) variant is the most frequently described pathogenic variant in *ALG13*, with 59 females and 2 males with this variant reported to date.

**Methods:**

We report on a male with a de novo, hemizygous variant in *ALG13*: c.320A>G; p.(Asn107Ser), whose phenotype resembles that of two previously reported males with the same variant.

**Results:**

All three males have a de novo mutation, infantile spasms, DEE, drug‐resistant epilepsy, intellectual disability, dysmorphic findings, recurrent infections, skeletal anomalies, brain abnormalities and a movement disorder: a phenotype not consistently reported in males with other pathogenic variants in *ALG13*.

**Conclusion:**

The similarity of phenotype in the three males with the c.320A>G variant in *ALG13*, suggests a possible genotype–phenotype correlation.

## Graphical abstract

We report on a male with a de novo, hemizygous variant in ALG13: c.320A>G; p.(Asn107Ser), whose phenotype resembles that of two previously reported males with the same variant. All have a de novo mutation, infantile spasms, developmental and epileptic encephalopathy, drug‐resistant epilepsy, intellectual disability, dysmorphic findings, recurrent infections, skeletal anomalies, brain abnormalities and a movement disorder: a phenotype not consistently reported in males with other pathogenic variants in ALG13.

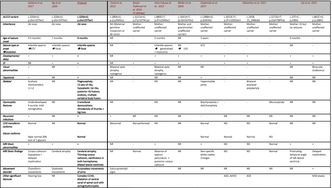

## 1 Introduction

Congenital disorders of glycosylation (CDG) are a group of neurometabolic disorders that result from genetic defects in the glycosylation of proteins and/or lipids (Timal et al. 2012). Pathogenic variants resulting in defects of N‐linked protein glycosylation were formerly known as CDG type 1 (CDG‐1) but are now written with the gene symbol followed by ‐CDG (Jaeken et al. 2009). ALG13‐CDG is a rare X‐linked form of this multisystem disorder (Timal et al. 2012; Ng et al. 2015), most of which are inherited as autosomal recessive traits (Hamici, Bastaki, and Khalifa 2017).

We report on a male with a de novo pathogenic hemizygous variant in the asparagine‐linked glycosylation 13 homolog (*ALG13*, OMIM: 300776) gene. ALG13 heterodimerises with asparagine‐linked glycosylation 14 homolog (ALG14) to form a complex in the endoplasmic reticulum that catalyses the second step in protein N‐glycosylation (Averbeck, Keppler‐Ross, and Dean 2007), a process critical for both the structure and function of these glycoproteins (Averbeck, Keppler‐Ross, and Dean 2007; Galama et al. 2017). The NM_001099922.3: c.320A>G; p.(Asn107Ser) variant is the most frequently described pathogenic variant in *ALG13* (Galama et al. 2017; NCBI 2022). It occurs in a highly functional position of the glycotransferase activity domain, which is important in the catalytic activity of the protein ALG13 (Galama et al. 2017; Hamici, Bastaki, and Khalifa 2017; Smith‐Packard, Myers, and Williams 2015). Because of its essential role in glycosylation, pathogenic variants in *ALG13* cause a congenital disorder of glycosylation named ALG13‐CDG, which has an X‐linked pattern of inheritance (Ng et al. 2020).

The NM_001099922.3: c.320A>G; p.(Asn107Ser) variant has been reported to date in 59 affected females and was initially assumed to cause embryonic lethality in males (Smith‐Packard, Myers, and Williams 2015). Most female patients with this variant present with developmental delay and epileptic spasms in the first year of life, consistent with a developmental and epileptic encephalopathy (DEE) phenotype (Datta et al. 2021). Intellectual disability, seizures and hypotonia have been reported, and approximately half of affected females have evolution of their seizures into other seizure types. Additional findings include ocular defects, craniofacial dysmorphism and gastrointestinal, cardiac, respiratory and immunological abnormalities. Transferrin isoelectric focusing or mass spectrometry was carried out in 16 of the reported female patients and all had glycosylation within the normal range (Ng et al. 2020; Alsharhan et al. 2021).

Currently there are two reported males with the NM 001099922.3: C.320A>G; p.(Asn107Ser) variant and 10 reported male patients with other pathogenic variants in *ALG13*. The first liveborn male with the NM 001099922.3: C.320A>G; p.(Asn107Ser) variant was reported by Galama et al. (2017). A second affected male was reported by Ng et al. (2020). Initially, both patients presented with infantile spasms and developmental delay. One progressed to drug‐resistant epilepsy (Galama et al. 2017), while the second has responded to levetiracetam following treatment with multiple anti‐seizure medications (ASMs) (Ng et al. 2020). Both have intellectual disability (ID) and other systemic features.

We report on a third male with the NM 001099922.3: C.320A>G; p.(Asn107Ser) variant in *ALG13*. His phenotype includes critical congenital heart disease (CCHD), severe global developmental delay, hypotonia, infantile spasms, focal seizures, skeletal anomalies, brain MRI abnormalities, dysmorphic craniofacial findings and a movement disorder.

## 2 Materials and Methods

### 2.1 Ethical Compliance

Written informed consent was provided by the parents of the proband (a minor), as per ethics protocol Study Code GEN/508/16, approved by the Ethics (Medical Research) Committee of Children's Health Ireland (CHI) at Crumlin, Dublin, Ireland.

The proband was enrolled in the PISCES Lighthouse Epilepsy Project to determine if an underlying genetic cause for his epilepsy and developmental delay could be detected (Benson et al. 2020). Trio exome sequencing and confirmatory Sanger sequencing were carried out as described in Benson et al. (2020). Variants were classified using the American College of Medical Genetics and Genomics (ACMG) guidelines (Richards et al. 2015).

## 3 Results

### 3.1 Clinical Report

The proband, a 12‐year‐old male, was born at term to 30+ year old non‐consanguineous healthy parents, following an uneventful pregnancy. His birth weight was 2.72 kg (10th centile). He has a younger brother who had a large patent ductus arteriosus requiring surgery at birth, but otherwise, the family history is non‐contributory.

At 6 weeks of age, the proband was diagnosed with critical cardiac anomalies, including aortopulmonary window, ventricular septal defect and anomalous origin of the right coronary artery from the pulmonary artery. He underwent corrective cardiac surgery at 3 months of age and his cardiac status has been stable since that time. As an infant, he had feeding difficulties and failure to thrive. A percutaneous gastrostomy tube, inserted at 3 years of age, is still in place as he continues to struggle with oral feeding. Developmentally he appears to be arrested at about 5 months of age and rolling over is the only motor developmental milestone that he has achieved. He is non‐verbal and has severe ID.

At 9 months of age, the proband was diagnosed with infantile spasms, which were difficult to control and required two courses of oral prednisolone. At approximately 23 months, he developed focal seizures which occurred in clusters. He has been treated with a number of ASMs (sodium valproate, levetiracetam, topiramate, clobazam and oxcarbazepine) but continues to have drug‐resistant epilepsy.

When he was an infant, there were no concerns regarding infections but in more recent years the proband has suffered from severe infections, including empyema and extensive staphylococcus infection at the PEG site. He does not have a bleeding disorder. No visual or hearing abnormalities have been detected.

Physical examination of the proband at 7 years of age showed that he was symmetrically small (0.4th centile for height and weight). His head circumference was <2SD. Dysmorphic findings included plagiocephaly, an irregular anterior hairline, relative telecanthus, wide palpebral fissures, a short nose, high nasal bridge, relatively long and flat philtrum, open mouth with tented upper lip and normal ear structure with mild bilateral posterior rotation. His hands were small (<3rd centile) with clinodactyly of the distal phalanx of both thumbs. His feet were small (<3rd centile) with clinodactyly of the distal phalanx of both 1st toes.

Neurological examination of the wheelchair‐bound proband revealed central hypotonia with evidence of hypertonia in his upper limbs. Constant purposeless movements of his upper limbs were observed.

### 3.2 Investigations

At 14 months of age, a Skeletal Survey showed 13 pairs of ribs, hypoplastic 1st ribs, fusion of posterior aspects of 11th and 12th ribs and narrowing of the T11/12‐disc space with a central area of fusion. There were also segmentation anomalies evident in the lumbar spine with partial fusion of the vertebral bodies at L3‐4 and L5‐S1 with associated disc space narrowing. The sacrum was short with fusion of S3‐4 segments. No other skeletal anomalies were noted.

At 7 years of age, a Brain MRI with MR Spectroscopy and Spinal MRI scans were carried out. Brain MRI showed cerebral atrophy, prominence of extra‐axial CSF spaces with mild‐to‐moderate dilated lateral ventricles, subtle increased signal intensity in the occiptal regions bilaterally, and many foci of calcified haemorrhage in the white matter. The corpus callosum was thin but normal in shape, and the cerebellum and brainstem were normal. MR Spectroscopy was normal. Spinal MRI revealed a small syrinx in the lower cervical cord and a large syringohydromyelia with septations in the thoracic cord. Several block vertebrae in the thoracic and lumbar regions and partial sacral agenesis were also noted (supplementary images).

At 9 months of age, his EEG showed typical hypsarrhythmia and epileptic spasms. His later EEGs, at 17 months and 3 years of age, showed a slow background with independent discharges from both hemispheres, with subclinical focal seizure arising from right posterior quadrant (supplementary images).

Metabolic Investigations: An extensive metabolic workup was carried out, including transferrin isoforms and APO3 isoforms, and all were reported as normal.

Genetics: Trio Exome sequencing revealed a de novo, hemizygous mutation in *ALG13*: NM_001099922.3:c.320A>G; p.(Asn107Ser)—ClinVar record (accession number SCV001160794.1), confirming the molecular diagnosis of ALG13‐CDG. This variant was classified as pathogenic (Evidence: PS1, PS2, PM2 and PP3).

## 4 Discussion

We report on a male with developmental and epileptic encephalopathy 36 (DEE36) with ID, the third reported male in the literature to date with the most common pathogenic variant in *ALG13*: c.320A>G; p.(Asn107Ser). The proband's phenotype is very similar to two previously reported males with the same pathogenic variant (Table 1) and despite the limited clinical information on the patient of Ng et al. (2020), all three individuals show a consistent pattern of clinical findings. They all presented within the first year of life with infantile spasms and associated developmental delay, which progressed to DEE36 with ID. All had drug‐resistant epilepsy, although one later responded to levetiracetam. Skeletal anomalies, brain abnormalities, dysmorphism and a movement disorder were also reported in all three males. Unsurprisingly, while similar systemic findings have been reported in some affected females, males with the same variant have a more severe phenotype (Table 2).

**TABLE 1:** Clinical findings and review of reported males with pathogenic variants in *ALG13*: Three patients with c.320A>G; p.(Asn107Ser) variant and 13 patients with 10 other variants.

	Galama et al. (2017)	Ng et al. (2020)	Proband	Timal et al. (2012)	Bissar‐Tadmouri et al. (2014) (4 siblings)	Hino‐Fukuyo et al. (2015)	Møller et al. (2016)	Gadomski et al. (2017)	Alsharhan et al. (2021)			Cai et al. (2022)	
*ALG13* variant	c.320A>G; p.(Asn107Ser)	c.320A>G; p.(Asn107Ser)	c.320A>G; p.(Asn107Ser)	c.280A>G; p.(Lys94Glu)	c.3221A>G; p.(Tyr1074Cys)	c.880C>T; p.(Pro294Ser)	c.1641A>T; p(.Gln547His)	c.1388A>G; p.(E463G)	c.3013C>T; p.(Pro1005Ser)	c.2458‐15_2486del	c.2272G>T; p.(Val758Phe)	c.23 T>C; p.(V8A)	c.862C>G; p.(L288V)
Inheritance	De novo	De novo	De novo	NK (Maternal germline mosaicism or de novo)	Mother: unaffected carrier	Mother: unaffected carrier	Mother and grandmother: unaffected carriers	Mother: unaffected carrier	Mother: unaffected carrier	Mother: unaffected carrier	Mother: unaffected carrier	Mother: ID but no seizures	Mother: unaffected carrier
Age of seizure onset	4.5 months	7 months	9 months	NR	−	5 months	NR	3 years	−	−	−	9 months	−
Seizure type at onset evolution	Infantile spasms GTC	Infantile spasms focal	Infantile spasms focal	NR	−	Infantile spasms generalized + myoclonic	NR LGS	GTC	−	−	−	NR	−
Developmental delay	+	+	+	NR	−	+	+	+	+	+	−	+	+
ID	NR	+	+	NR	+	+	+	−	+	+	−	+	+
Visual abnormalities	+	NR	−	Bilateral optic atrophy, nystagmus	−	Bilateral optic atrophy, nystagmus	NR	NR	−	−	−	NR	Binocular strabismus
Hypotonia	+	NR	+	NR	−	NR	NR	+	−	−	−	NR	NR
Skeletal	Scoliosis Hemivertebra L1‐L2	NR	Plagiocephaly; 13 sets of ribs, hypoplastic 1st ribs, posterior rib fusions; scoliosis, multiple vertebral body fusion	NR	−	NR	NR	Hypermobile joints	−	Bilateral postaxial polydactyly	−	NR	NR
Dysmorphic features	Underdeveloped R auricle; mild retrognathia	NR	Craniofacial dysmorphism; clinodactyly of thumbs + big toes	NR	−	NR	NR	Brachycephaly + dolichocephaly	−	−	Microcephaly	NR	NR
Recurrent infections	+	NR	+	+	NR	NR	NR	−	NR	NR	NR	NR	NR
CDG transferrin isoforms Glycan isoforms	Normal Near normal (6% lack of 1 glycan)	NR	Normal Normal	Abnormal	Not performed	NR	NR	Normal Normal	ND Normal	ND Normal	Normal ND	NR	NR
MRI Brain abnormalities	+	+	+	NR	−	+	NR	+	ND	ND	Normal	+	+
MRI Brain findings	Corpus callosum hypoplasia + delayed myelination	Cerebral atrophy	Cerebral atrophy; Thinning corpus callosum; calcification in both hemispheres; dilated lateral ventricles	NR	Normal	Absence of septum pellucidum + posterior corpus callosum	NR	Non‐specific white matter changes	ND	ND	Normal	Protruding temporal angle of left lateral ventricle	Delayed myelination
Movement disorder	Choreiform movements	Dyskinetic movements	Purposeless movements of arms	Extra‐pyramidal signs	−	−	NR	NR	NR	NR	NR	NR	+
Other significant features	Hearing loss	NR	Complex CCHD; dilatation of central canal of spinal cord with syringohydromyelia						ASD; ADHD	ASD			Mild ataxia

Abbreviations: CCHD: critical congenital heart disease; GTC: generalized tonic–clonic; ID: intellectual disability; ND: not done; NK: not known; NR: not reported.

**TABLE 2:** Phenotype of male and female patients with c.320A>G; p.(Asn107Ser) variant in *ALG13*.

	Total female cases = 59	Total male cases = 3
Inheritance	De novo = 55 (NR = 4)	De novo = 3
Age of seizure onset	1–18 months (1 at 13 years)	4.5–9 months
Infantile spasms	49/59	3/3
Seizure type at onset evolution over time	Infantile spasms (49/56) Polymorphic seizures 2; GTC 5; tonic 5; myoclonic 3; focal 4; LGS 8; absence 2	Infantile spasms (3/3) GTC: 1; Focal: 2
Developmental delay	57/59	3/3
ID	48/59	2/3 (NR = 1)
Visual abnormalities	12/59	1/2 (NR = 1)
Hypotonia	13/59	2/3
Skeletal anomalies	2/59	2/3 (NR = 1)
Dysmorphic features	11/59	2/3 (NR = 1)
Skull	Brachycephaly: 2 Microcephaly: 2	Plagiocephaly (1/2) NR = 1
Facial dysmorphisms	Hypertelorism (2/59) Broad coarse face Low set ears (1/59) Micrognathia/retrognathia (3/59) Turned‐up nose (1/59) Long philtrum (1/59) Synophrys (1/59) Long eyelashes (1/59) Widely spaced teeth (1/59) Enophthalmos + hypotelorism (2/59) Anteverted nares (1/59)	Multiple craniofacial dysmorphisms (1/3) Irregular anterior hairline Relative telecanthus Wide palpebral fissures Short nose High nasal bridge Long and flat philtrum Open mouth + tented upper lip Underdeveloped R auricle + mild retrognathia (1/3) NR = 1
Hands + Feet	Small hands (1/59) Adducted thumbs (1/59) Bilateral clinodactyly + syndactyly of toes (1/59)	Clinodactyly of thumbs + big toes (1/3) Small hands (1/3) NR = 1
Recurrent infections	0/59	2/3
CDG transferrin isoforms Glycan isoforms	Normal 16/59 (NR = 43) Reported in two cases as moderate changes	Normal 2/3* (NR = 1) *Normal: 1; Near normal: 1
MRI Brain abnormalities	14/59	3/3
Movement disorder	18/59	3/3

Abbreviations: GTC: generalized tonic–clonic; LGS: Lennox Gastaut Syndrome; NR: not reported.

In other reported males with different pathogenic variants in *ALG13*, the typical phenotype of DEE36 was not consistently displayed (Table 1). For example, in one family of four affected brothers with the NM_001099922.3:c.3221A>G; p.(Tyr1074Cys) variant in *ALG13*, epileptic seizures or the other described phenotypic changes did not occur, and this family presented with non‐syndromic X‐linked intellectual disability (NSXLID) (Bissar‐Tadmouri et al. 2014). Furthermore, a male with the NM_001099922.3:c.1388A>G; p.(E463G) variant developed seizures at 3 years of age which were well controlled on one ASM (Gadomski et al. 2017).

While congenital heart disease (CHD) has been reported in approximately 20% of individuals with ALG13‐CDG (Marques‐da‐Silva et al. 2017), the proband has a family history of a sibling who had a large patent ductus arteriosus detected at birth. Therefore, the occurrence of CHD in the proband must be interpreted with caution in this family as this may have a separate aetiology. A pathogenic variant for CHD was not detected on exome sequencing but since the majority of patients with CHD do not have a molecular diagnosis, this is not an unusual finding (Diab et al. 2021).

There are 59 reported females with the NM 001099922.3: c.320A>G; p.(Asn107Ser) variant, 55 of whom have a de novo heterozygous variant (de Ligt et al. 2012; Allen et al. 2013; Michaud et al. 2014; Dimassi et al. 2016; Kobayashi et al. 2016; Ortega‐Moreno et al. 2017; Zhu et al. 2017; Palmer et al. 2018; Yuskaitis et al. 2018; The Deciphering Developmental Disorders Study 2018; Fung et al. 2017; Geisheker et al. 2017 and Madaan et al. 2019). The mode of inheritance in four individuals is not recorded. Three affected females with a heterozygous pathogenic variant in *ALG13* had X chromosome inactivation (XCI) analysis performed and random X inactivation was reported from whole blood samples in all three individuals (Ng et al. 2020). This is a very small sample of affected females, but the result is consistent with X‐linked dominant inheritance of ALG13‐CDG in these females.

The affected males with the NM 001099922.3: c.320A>G; p.(Asn107Ser) variant all have a de novo variant but the 13 reported males with other pathogenic variants in *ALG13* all have maternal inheritance of the variant (Table 1). Because the report of Bissar‐Tadmouri et al. (2014) includes four affected male siblings, Table 1 shows 13 affected males who inherited 10 individual pathogenic variants in *ALG13*. Nine affected males inherited the variant from an unaffected carrier mother while the mother of one affected male had ID but no seizures (Cai et al. 2022). In their report of two of the affected males, Cai et al. (2022) suggested that the inheritance in the male with the affected mother was X‐linked dominant and in the male with the unaffected mother, the inheritance was X‐linked recessive. This would be very unusual and cannot be verified without XCI analysis of both mothers in this study (Cai et al. 2022). X‐inactivation analysis was not carried out on any of the 10 carrier mothers but non‐random X inactivation in nine of the mothers may be one explanation for their unaffected status, with random X inactivation, or a separate disorder, resulting in the phenotype of the affected mother.

In conclusion, the phenotype of the proband with the NM 001099922.3: c.320A>G; p.(Asn107Ser) variant in *ALG13* is very similar to the two previously reported male patients with a similar variant, a pattern which has not been consistently reported in males with other pathogenic variants in *ALG13*. The similarity of phenotype in the three males with the NM 001099922.3: c.320A>G; p.(Asn107Ser) variant in *ALG13*, suggests a possible genotype–phenotype correlation.

## Author Contributions

The concept for the article was proposed by Marie T. Greally. The original draft was written by Rebecca Finnegan and reviewed and edited by Marie T. Greally and Rebecca Finnegan, who also collected the relevant patient data, designed and formatted the Tables, and carried out a literature review. Máire White obtained the initial clinical information from the parents of the proband prior to his inclusion in the PISCES Lighthouse Project. The exome analysis results were reviewed and confirmed by Katherine A. Benson and Gianpiero L. Cavalleri, who also contributed to the writing and editing of the article. Mary O'Regan provided clinical information on the proband and Norman Delanty contributed to the writing and editing of the article. All authors read and approved the final manuscript.

## Conflicts of Interest

The authors declare no conflicts of interest.

## Supporting information


Figure S1.

Figure S2.

Figure S3.

## Data Availability

The data that support the findings of this study are available from the corresponding author upon reasonable request.
