# Supplementary material for: Similarity of Phenotype in Three Male Patients With the c.320A>G Variant in ALG13: Possible Genotype–Phenotype Correlation
Source: Mol Genet Genomic Med. 2024 Sep 23;12(9):e70010. doi: 10.1002/mgg3.70010 (PMC11418404; doi:10.1002/mgg3.70010)

**
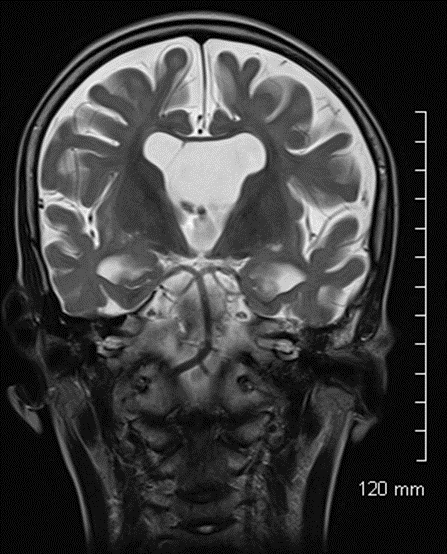

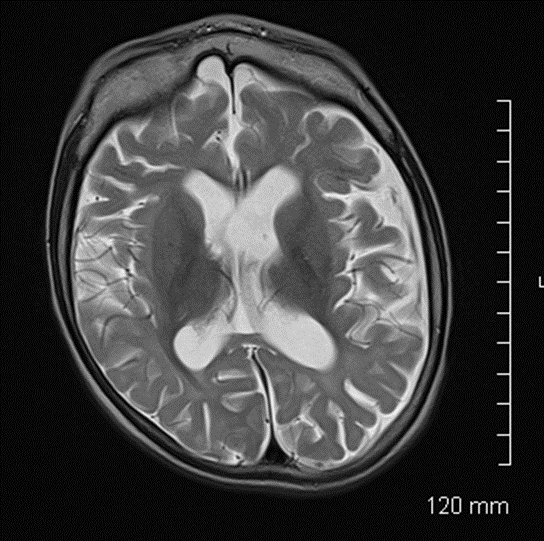
Supplementary Images 1 - MRI Brain (7 years old)**

C

B

A

Image A – Axial T2

Image B – Sagittal T1

Image C – Coronal T2

These images show cerebral atrophy, prominence of extra-axial CSF spaces with mild-to-moderate dilated lateral ventricles, subtle increased signal intensity in the occiptal regions bilaterally, and many foci of calcified haemorrhage in the white matter. The corpus callosum was thin but normal in shape. Normal cerebellum and brainstem.


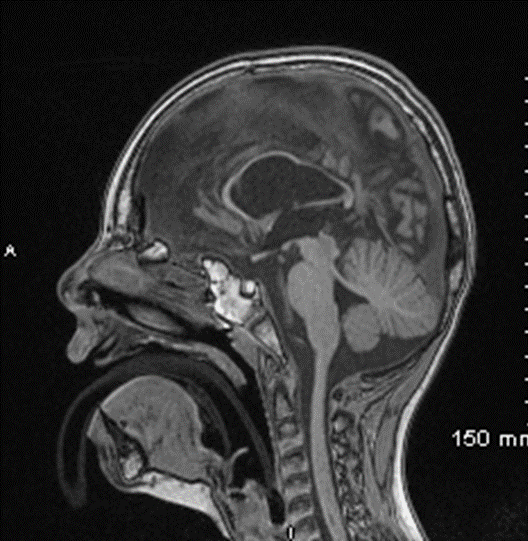

Supplement: Supplementary file 1 — Figure S1. [file MGG3-12-e70010-s001.docx]
