# Supplementary material for: Similarity of Phenotype in Three Male Patients With the c.320A>G Variant in ALG13: Possible Genotype–Phenotype Correlation
Source: Mol Genet Genomic Med. 2024 Sep 23;12(9):e70010. doi: 10.1002/mgg3.70010 (PMC11418404; doi:10.1002/mgg3.70010)
Supplement: Supplementary file 2 — Figure S2. [file MGG3-12-e70010-s002.docx]

**Supplementary Images 2 - MRI Spine (7 years old)**


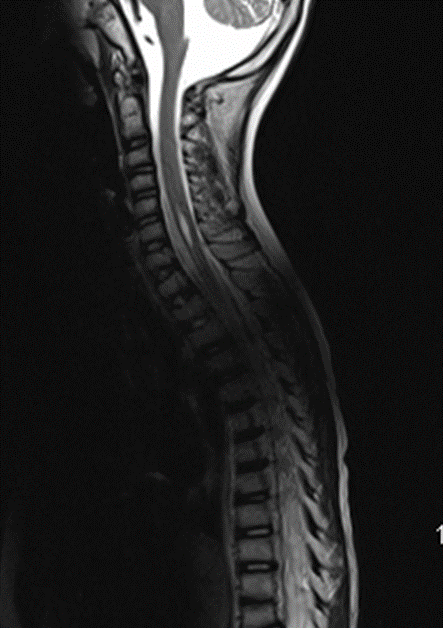

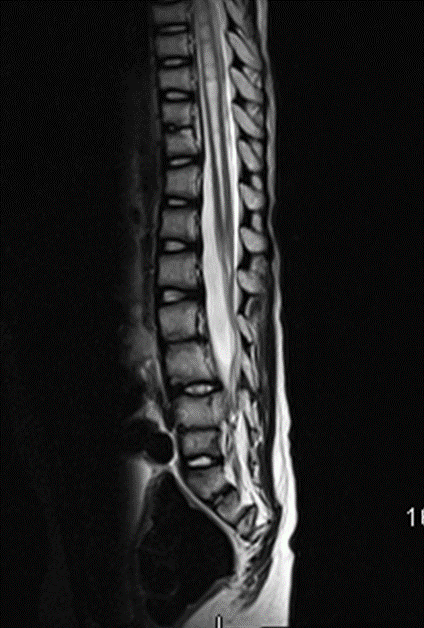

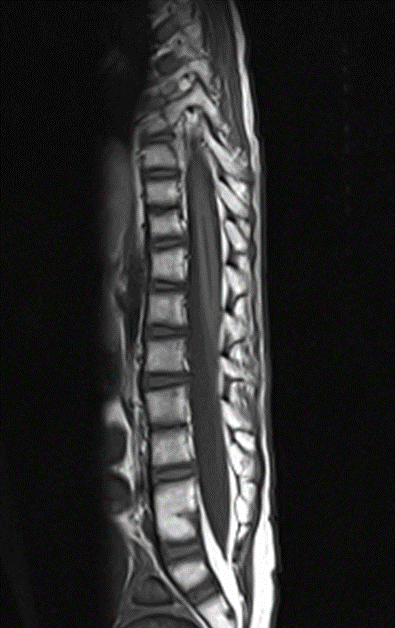


B

Image A – sagittal T2 upper spine

Image B – Sagittal T2 lower spine

Image C – Sagittal T1 lower spine

These images show a small syrinx in the lower cervical cord and a large syringohydromyelia with septations in the thoracic cord. Several block vertebrae in the thoracic and lumbar regions and partial sacral agenesis.

C

B

A
