# Supplementary material for: Similarity of Phenotype in Three Male Patients With the c.320A>G Variant in ALG13: Possible Genotype–Phenotype Correlation
Source: Mol Genet Genomic Med. 2024 Sep 23;12(9):e70010. doi: 10.1002/mgg3.70010 (PMC11418404; doi:10.1002/mgg3.70010)
Supplement: Supplementary file 3 — Figure S3. [file MGG3-12-e70010-s003.docx]

**Supplementary Images 1 – EEG recordings (17 months old and 3 years of age)**


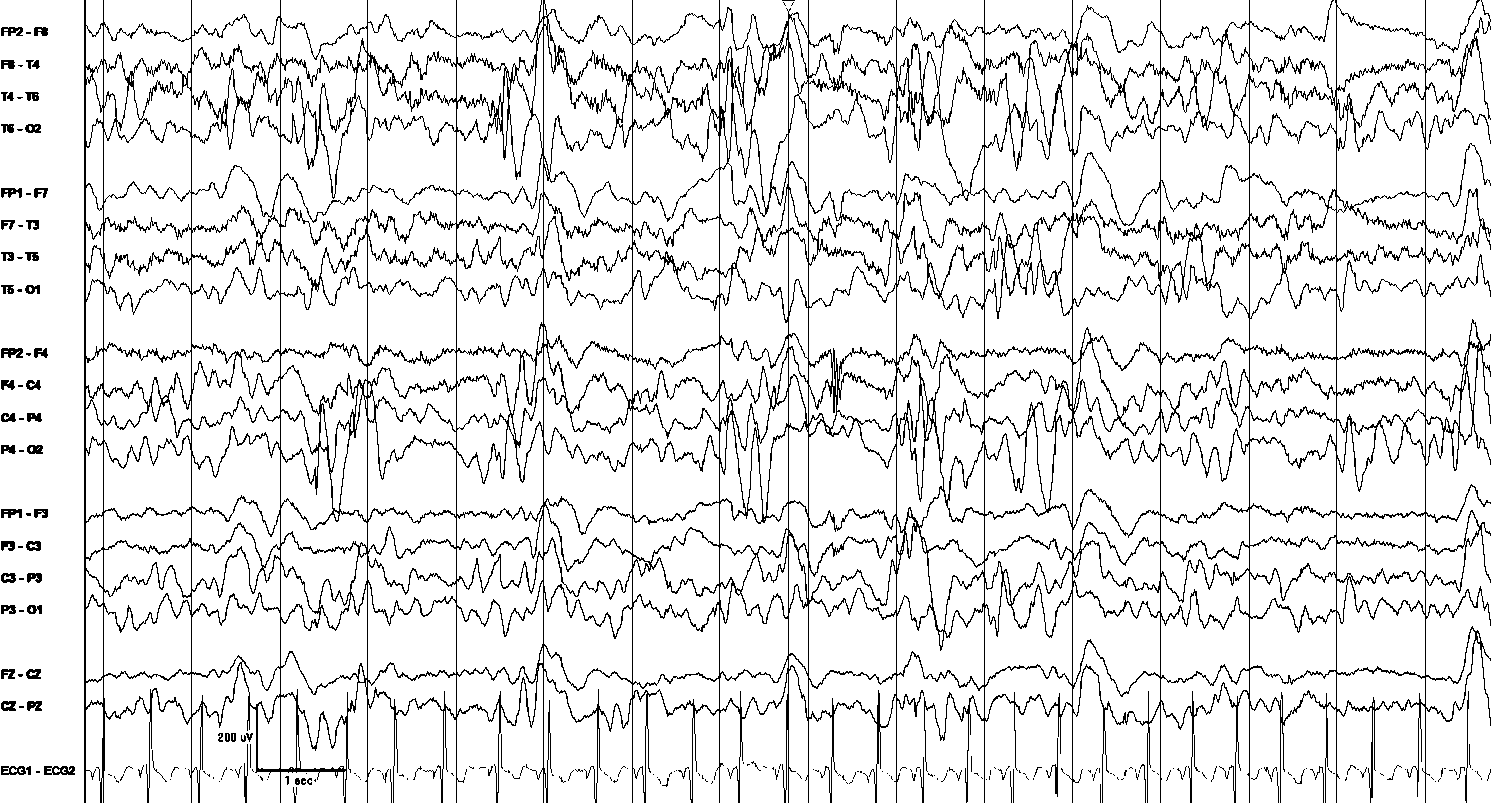


Image A: Independent right>left sided posterior quadrant discharges (age 17 months); LFF 0.5Hz, HFF 70Hz


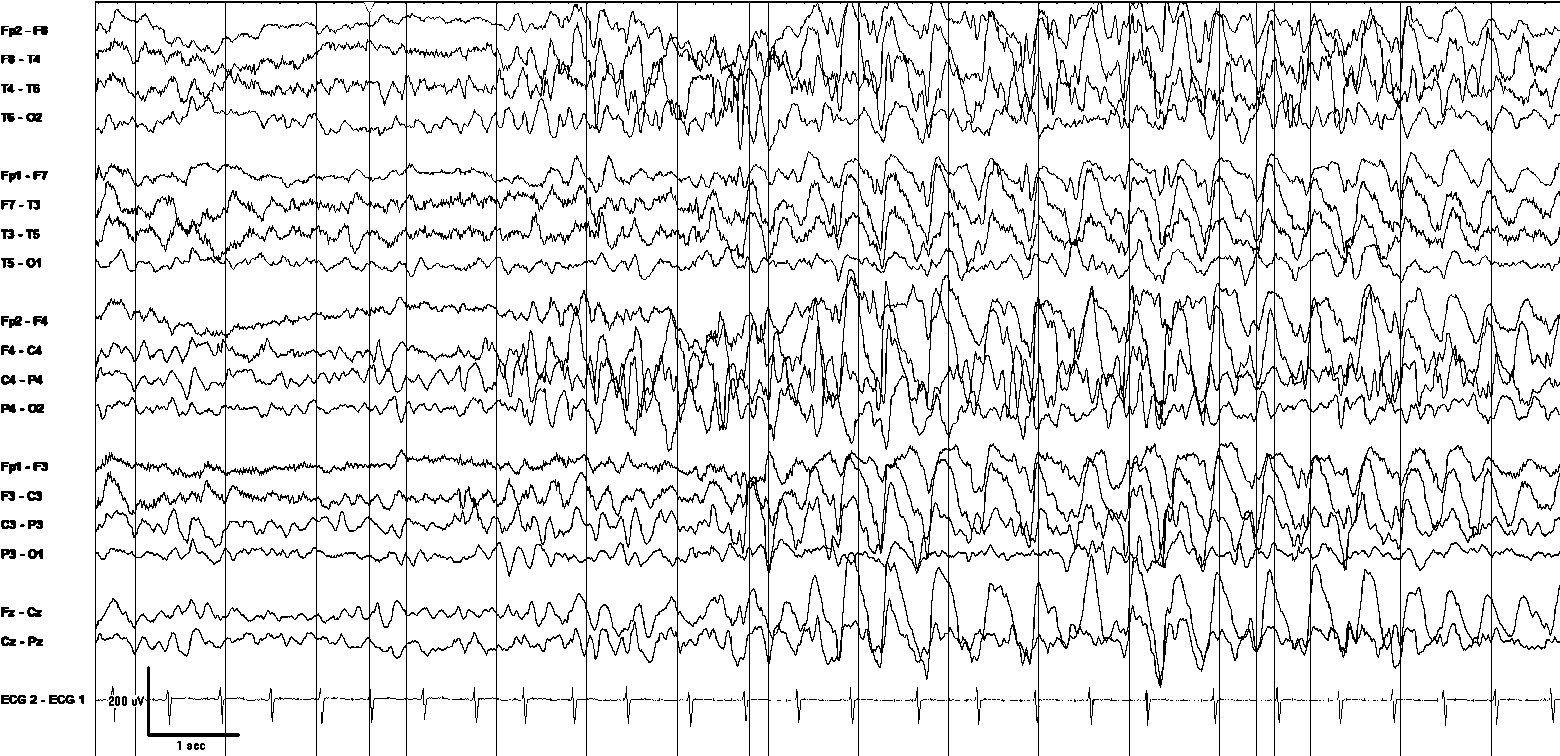


Subclinical focal seizure from right posterior quadrant (3yrs);
LFF 1.0Hz, HFF 70Hz
